# The Effect of Germination on Phenolic Content and Antioxidant Activity of Chickpea

**Published:** 2012

**Authors:** Babak Ghiassi Tarzi, Maryam Gharachorloo, Marzieh Baharinia, Seyed Alireza Mortazavi

**Affiliations:** a *College of Food Science and Technology, Science and Research Branch, Islamic Azad University, Tehran, Iran. *; b *Student of Food Science and Technology, Science and Research Branch, Islamic Azad University, Tehran, Iran. *; c *Pharmaceutics Department, School of Pharmacy, Shahid Beheshti University of Medical Sciences, Tehran, Iran.*

**Keywords:** Antioxidant activity, Chickpea, Germination, Phenolic compounds

## Abstract

Germination is one of the most effective processes to improve the quality of legumes. Vitamins and some other compounds that might be considered beneficial as antioxidants, often change dramatically during the course of germination. Antioxidants might be defined as compounds which are capable of preventing, delaying or retarding the development of rancidity or other flavor deterioration in foods or as protective factors against the oxidative damage in the human body. In this research, three different solvents were employed to extract the phenolic compounds present in chickpea seeds and sprouts. Total phenolic contents were measured by Folin Ciocalteau method and the antioxidant activity was determined by two different methods including the assay of hydroxyl radical scavenging activity and the oven test method. For the later, different concentrations of extracts (0.02, 0.04, 0.06, 0.08 and 0.1% w/w) were added to tallow and the stabilities of the treatments were determined. Peroxide value and induction period measurements were used as means to evaluate the antioxidant activities. The results indicated that germination process modifies the antioxidant activity. Although the amount of phenolic compounds was higher when acetone solvent was employed, methanolic extract indicated better hydroxyl radical scavenging activity. The evaluation of the antioxidant activity of the extracts activity was concentration-dependent by delaying the indicated oxidation and increased when higher concentrations of the extracts were applied. Therefore, chickpea sprout flour or extract might be used as a source of natural antioxidants in functional foods or in the formulation of the oil-based supplements or medicine in the form of capsule.

## Introduction

Legumes, one of the most important sources of food in the world, play an important role in human nutrition in many countries (1). Some biotechnological processes and methods such as germination are considered both simple and economical to improve the nutritive value of legumes by causing desirable changes in the nutrient availability, texture and organoleptic characteristics (2). Extensive breakdown of seed-storage compounds and synthesis of structural proteins and other cell components take place during the germination. Vitamins and secondary compounds, many of which are considered beneficial as antioxidants, often change dramatically during the germination (3). It is known that the germination process generally improves the nutritional quality of legumes, not only by the reduction of antinutritive compounds, but by increasing the levels of free amino acids, available carbohydrates, dietary fiber, and other components, and also increasing the functionality of the seeds due to the subsequent increase in the bioactive compounds (4).

One of these bioactive compounds are polyphenols which are quite suitable for protecting cell membranes against the damage induced by reactive free radicals and are able to reduce the LDL aggregation (5). Phenolic compounds not only effectively prevent the oxidation in foods, they also act as protective factors against oxidative damage in the human body (4). Epidemiological studies show that the consumption of food with high phenolic content is correlated with reduced cardiovascular, inflammation, cancer mortality

and some other disease rates (6). Fernandez-Orozco *et al*. (2) in 2009 investigated the effect of germination to improve the antioxidant properties of chickpeas. The results indicated that germination caused an increment in total phenolic content, increased peroxyl radical-trapping capacity (16-55%)

and trolox equivalent antioxidant capacity (TEAC) (12-23%) and a slight inhibition of lipid peroxidation inhibition was observed. Total phenolic compounds highly contributed to total antioxidant capacity. Results indicated that with the germination, the antioxidant properties of chickpea flours are enhanced and they can be used as desired ingredients for new functional food formulations. Cevallos-Casals and Cisneros-Zevallos (7) in 2010 analyzed 13 edible seeds for the levels of phenolic compounds and total antiradical capacity (TAC) at different germination states (dormant, imbibed and 7d sprouts). Accumulated phenolics (mg chlorogenic acid equivalent, CAE) and TAC (μg Trolox equivalent) on dry

basis (DB) showed the general trend distribution of 7d sprouts > dormant seeds > imbibed seeds.

Phenolic contents of 7d sprouts (DB) ranged from 490 (lentil) to 5676 (mustard) mg CAE 100 g-1. Increases in phenolics (DB) from dormant seed to 7d sprout differ among seeds from 2010% (mung bean) to -11% (kale), while increases in TAC (DB) ranged from 1928% (mung bean) to 0% (lentil). This study showed that germinated edible seeds are an excellent source of dietary phenolic antioxidants.

Lopez-Amoros *et al*. (4) in 2006 studied the effects of varying germination conditions for beans, lentils and peas, at semi-pilot scale, on bioactive compounds and expressed that peas and beans undergo a significant increase in antioxidant activity after germination, whereas lentils show a decrease. The present study was concerned and aimed at the evaluation and influence of the germination process on the phenolic content and antioxidant capacity of chickpea seeds in order to obtain suitable flour or extract with high nutritive value and antioxidant activity as an ingredient in supplements or medicine formulation.

## Experimental

Chickpea seeds were supplied from Tehran market. Seeds were cleaned and stored in darkness in polyethylene containers at 4°C. Mutton tallow was obtained by dry rendering of sheep’s tail fat under vacuum employing a rotary evaporator at 60 rpm and 80°C for 2 h. All the chemicals used were of analytical grade, purchased from Merck Chemical Company (Merck, Germany).

*Germination process*

Two hundred g of seeds were soaked in 1000 mL of 0.07% sodium hypochlorite for 30 min. These seeds were washed with distilled water until reaching to neutral pH and then were soaked with 1000 mL of distilled water for 5 h, shaking every 30 min. The hydrated seeds were located in germination trays on wet laboratory paper and covered, where water circulation by capillarity was created. The trays were

introduced in the germination machine IKHRH model. The seeds were germinated at 20°C, 99% relative humidity in darkness for 5 days. The maximum time of germination was fixed in accordance with achieving 95% sprout seeds. The germination process was evaluated by the percentage of germinated seeds. The sprouted seeds were collected, grounded in the mill (Triplex, France), passed through a sieve of 0.5 mm and the obtained flour was stored in plastic bags, in darkness at 4°C (4). A blank consisted of ungerminated grains flour were also prepared.

*Preparation of phenolic extracts*

Flour of chickpea seeds and chickpea sprouts were subjected to the extraction of phenolic compounds using different solvents (methanol, acetone and hexane) individually and stirred for 24 h at room temperature. The extracts were centrifuged at 4000 rpm for 15 min and filtered through the filter paper (Whatman NO. 41). The solvents were removed using a rotary evaporator at 40ºC under vacuum. The extracts were dried using vacuum oven at 40ºC and were kept in dry clean black glass bottle at 4ºC for further analysis.

*Assay of total phenolic compounds*

Total phenolic compounds were determined according to Fernandez-Orozco *et al*. (5) in 2006. The method is based on the color reaction of Folin-Ciocalteu reagent with hydroxyl groups. Absorbance was measured at 765 nm using a spectrophotometer (Optizen 2120, South Korea). The results were expressed as mg Gallic acid per Kg of extract.

*Assay of hydroxyl radical (OH–) scavenging activity*

The assay was based on the benzoic acid hydroxylation modified method, as described by Chung *et al. *(8) in 1997 and Bahramiyan *et al. *(9) in 2012. In a screw-capped tube, 1 mL of sodium benzoate, (50 mmol) and 1 mL of FeSO4.7H2O (50 mmol) and EDTA (50 mmol) were placed. Phosphate buffer (pH = 7; 0.1 mol) was added to the sample solution to give a total volume of 9 mL. Finally, 1 mL of a H2O2 solution (50 mmol) was added. The mixture was incubated at 37°C for 2 h and the absorbance was measured at 610 nm where the OH-scavenging activity might be expressed as follows:

$\mathrm{Scavenging}\left(\%\right)=\frac{\left(A_{{}^{\circ}}-A_{s}\right)}{A_{{}^{\circ}}}$

Here, *A° = *absorbance for control and *As = *absorbance for sample.

*Assay of antioxidant activity by the oven test method (* 10 *,* 11 *)*

Mutton tallow which might contain minor natural antioxidants was used as a basic substrate for the evaluation of antioxidant activity of chickpea sprout extracts. The collected hexane, acetone and methanolic extracts were added to 100 g of mutton tallow at the concentrations of 0.02, 0.04, 0.06, 0.08 and 0.1% (w/w) to examine their antioxidant activity. Induction period measurements for each treatment were performed on Metrohm Rancimat model 743 at 110°C with airflow of 20 L/h. Peroxide value determinations were carried out by placing the treatments in the oven at 90ºC and measuring the peroxides every 24, 48, 72, 96 and 120 h according to AOAC official method (Method 965.33) (12).

*Statistical analysis*

All the experiments and measurements were carried out in triplicate order. The data were statistically analyzed using the Statistical Analysis System software package on replicated test data. Analyses of variance were performed by the application of ANOVA procedure. Significant differences between the means were determined using Duncan multiple range test.

## Results and Discussion

The total phenolic content in hexane, acetone and methanolic extracts of chickpea seeds and chickpea sprouts are shown in Table 1: The highest extraction rate of phenolic compounds in chickpea seeds was obtained by acetone solvent. 

**Table 1:** Total phenolic content of hexane, acetone and methanolic extracts of chickpea seeds and chickpea sprouts (mg/Kg).

**Sample/solvent**	**Hexane**	**Methanol**	**Acetone**
Chickpea	41.78 ± 0.06a*	39.20 ± 0.10b	126.00 ± 0.05c
Germinated chickpea	66.90 ± 0.15d	75.60 ± 0.24e	193.70 ± 0.00f

*The values are expressed as mean ± standard deviation, n = 3. There are no significant differences between similar letters (p < 0.05).

Lopez-Amoros *et al*. (4) in 2006 indicated that chickpeas contain different concentrations of hydroxybenzoic phenolic compounds, protocatechuic, p-hydroxybenzoic, vanillic acid, trans-ferulic acid, cis and trans p-coumaric acid. During the germination, the amount of phenolic compounds was increased. Increase in extraction rate of total phenolic compounds of acetone, hexane and methanolic extracts of germinated chickpeas, as compared with seeds, were 53.7, 60.1 and 92.8 % respectively. Lopez-Amoros *et al*. (4) in 2006 indicated that germination modifies the quantitative and qualitative phenolic compounds of legumes, and the changes depend on the type of legume and the germination conditions. These changes influence the functional properties of the legumes as the consequence of variation in antioxidant activity. Acetone and methanolic extracts of chickpea seeds indicated the highest (78%) and lowest (15%) antioxidant activities respectively, which might be due to the difference in solvents polarity and consequently the type of extracted compounds (Table 2).

**Table 2:** Antioxidant Activity of hexane, acetone and methanolic extracts of chickpea and chickpea sprouts (*%*).

**Sample/solvent**	**Hexane**	**Methanol**	**Acetone**
Chickpea	71 ± 0.5a*	15 ± 1.0b	78 ± 0.0c
Germinated chickpea	80 ± 0.0c	89 ± 0.5d	81 ± 0.5c

* The values are expressed as means ± standard deviation, n = 3. There is no significant difference between similar letters (p < 0.05).

**Figure 1:**
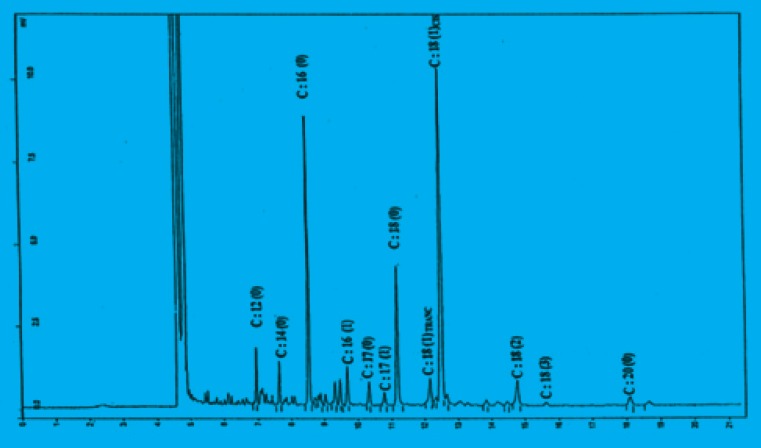
Fatty acid profile of mutton tallow

 There is significant difference between acetone, hexane and methanolic extracts (p < 0.05). The results indicated the significant increase in antioxidant activity of chickpea seeds after the germination. Although the methanolic extract of chickpea seed showed the lowest antioxidant activity, after the germination, it has the best antioxidant activity. Although acetone extracts contained the highest total phenolic compounds, the results indicated that methanolic extracts has the highest antioxidant activity. Therefore, it seems that additional antioxidants other than polyphenols might be present in extracts. Legumes contain other bioactive compounds beside phenolic such as vitamins and carotenoids at different concentrations that might also behave as antioxidant (1, 13). These compounds might also exert synergetic activities among themselves and with phenolic compounds, which could be the main reason of the observed differences in

the antioxidant activities. Fernandez-Orozco *et al*. (5) in 2006 indicated that germinated lupins provided more vitamin C, vitamin E activity and polyphenols than raw seeds, and the largest amounts of these bioactive compounds were found after 6 days of germination. Therefore, they stated that the germination of lupin seeds (*Lupinus* *angustifolius *L. var. Zapaton) might be a good process to enhance their antioxidant capacity. Mutton tallow which contains minor natural antioxidants was used as a basic medium for the evaluation of antioxidant activity of chickpea sprout extracts by the method of delay in fat oxidation. Figure 1 shows the gas chromatogram of tallow which contains 49.3% and 46.4% of unsaturated and saturated fatty acid respectively. Peroxide values of tallow without phenolic extracts have been increased through the oxidation as the time of heating is increased. 

Table 3 shows the peroxide values of tallow treated with different concentrations of extracts. The results shown in Table 3 indicate that all the used concentrations of acetone, hexane and methanolic extracts of chickpea sprouts decreased the rate of tallow oxidation. Significant differences were found between all treatments (p < 0.05).

**Table 3:** Peroxide values of tallow with different concentrations of chickpea sprouts extracts at 90ºC (meq/Kg oil)a

**Treatment/Time of Heating**	**0 (h)**	**24 (h)**	**48 (h)**	**72 (h)**	**96 (h)**	**120 (h)**
**Control**	0.20 ± 0.00	3.65 ± 0.25	5.35 ± 0.25	6.45 ± 0.25	6.90 ± 0.00	8.65 ± 0.15
**Extract (0.02%) Hexane**	0.20 ± 0.00	3.25 ± 0.05	4.00 ± 0.10	3.90 ± 0.20	5.25 ± 0.45	6.35 ± 0.05
**Extract (0.04%) Hexane**	0.20 ± 0.00	2.55 ± 0.35	2.95 ± 0.05	3.90 ± 0.00	4.60 ± 0.10	5.75 ± 0.35
**Extract (0.06%) Hexane**	0.20 ± 0.00	1.90 ± 0.10	1.90 ± 0.10	3.05 ± 0.65	3.90 ± 0.70	4.75 ± 0.35
**Extract (0.08%) Hexane**	0.20 ± 0.00	2.40 ± 0.30	3.65 ± 0.15	4.00 ± 0.10	4.70 ± 0.20	6.50 ± 0.40
**Hexane Extract (0.1%)**	0.20 ± 0.00	1.95 ± 0.95	3.85 ± 0.15	4.35 ± 0.15	5.50 ± 0.20	6.55 ± 0.05
**Methanol Extract (0.02%)**	0.20 ± 0.00	2.45 ± 0.25	3.65 ± 0.25	4.35 ± 0.25	6.35 ± 0.15	7.75 ± 0.05
**Methanol Extract (0.04%)**	0.20 ± 0.00	2.35 ± 0.15	3.05 ± 0.15	5.25 ± 0.05	5.95 ± 0.15	7.20 ± 0.10
**Methanol Extract (0.06%)**	0.20 ± 0.00	2.20 ± 0.10	2.35 ± 0.25	3.65 ± 0.15	4.75 ± 0.05	6.15 ± 0.35
**Methanol Extract (0.08%)**	0.20 ± 0.00	1.50 ± 0.40	2.70 ± 0.20	3.85 ± 0.05	4.75 ± 0.05	5.10 ± 0.00
**Methanol Extract (0.1%)**	0.20 ± 0.00	1.45 ± 0.35	3.30 ± 0.20	4.45 ± 0.15	4.90 ± 0.40	5.30 ± 0.15
**Acetone Extract (0.02%)**	0.20 ± 0.00	1.25 ± 0.15	1.40 ± 0.00	2.85 ± 0.35	4.60 ± 0.10	5.35 ± 0.25
**Acetone Extract (0.04%)**	0.20 ± 0.00	0.80 ± 0.00	1.45 ± 0.05	2.10 ± 0.40	3.30 ± 0.05	3.90 ± 0.00
**Acetone Extract (0.06%)**	0.20 ± 0.00	1.00 ± 0.30	1.30 ± 0.5	1.50 ± 0.20	2.55 ± 0.35	3.50 ± 0.10
**Acetone Extract (0.08%)**	0.20 ± 0.00	0.30 ± 0.00	0.75 ± 0.15	1.65 ± 0.15	2.45 ± 0.05	3.00 ± 0.10
**Acetone Extract (0.1%)**	0.20 ± 0.00	0.20 ± 0.00	1.30 ± 0.05	3.00 ± 0.30	3.25 ± 0.25	4.55 ± 0.45

a: The values are expressed as means ± standard deviation, n = 3

Acetone extracts of chickpea sprouts showed lower rate of oxidation and consequently lower peroxide values were obtained. This might be due to the higher total phenolic compounds present in this extract as indicated in Table 1. The evaluation of the antioxidant activity of the extracts indicated that the activity was concentration-dependent and increased when higher concentrations of the extracts were applied.

**Figure 2:**
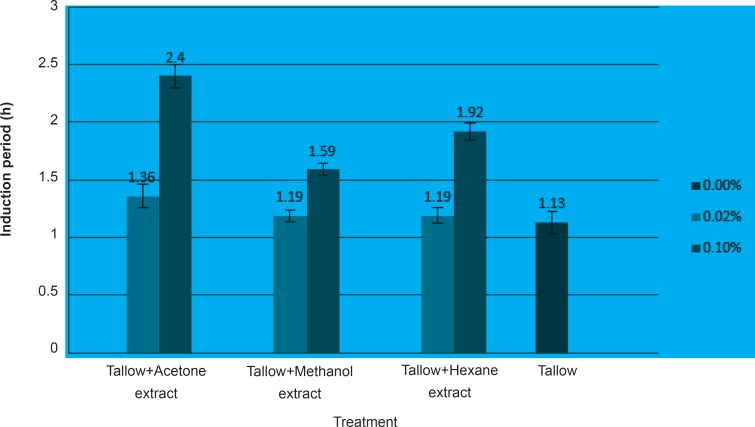
Induction period of tallow treated with 0.02 and 0.1% of chickpea sprout extracts

Although all the used germinated chickpea extracts reduced the formation of peroxide, 0.06% concentration of hexane and methanolic extracts and 0.08% concentration of acetone extract gave better results than the others. Figure 2 indicates the induction period of tallow treated with the minimum (0.02%) and maximum (0.1%) concentrations of chickpea sprout extracts.

 Acetone extracts increased the induction period of tallow higher than others. The results of the induction period which is related to the secondary oxidation products confirms the findings by peroxide values, the primary oxidation products that antioxidant activity was increased when higher concentrations of the extracts were applied.The results indicated that the germination process causes various changes in the phenolic compounds and modifies the antioxidant activity. Acetone extracts of chickpea sprouts which contained higher phenolic compounds showed better antioxidant activities. Since the source of some substances used in the pharmaceutical industry are secondary metabolites of plants such as phenolic compounds which nutritional and medicinal values of them has been demonstrated, chickpea sprout flour or extract might be used as a source of natural antioxidants in functional foods, supplements and some medicine formulations.

## References

[1] Prodanov M, Sierra I, Vidal-Valverde C (1998). Effect of the germination on the thiamine, riboflavin and niacin contents in legumes. Food Res. Technol.

[2] Fernandez-Orozco R, Frias J, Zielinski H, Munoz M, Piskula MK, Kozlowska H, Vidal-Valverde C (2009). Evaluation of bioprocesses to improve the antioxidant properties of chickchickpeas. Food Res. Technol.

[3] Kuo YH, Rozan P, Lambein F, Frias J, Vidal- Valverde C (2004). Effects of different germination conditions on the contents of free protein and non-protein amino acids of commercial legumes. Food Chem.

[4] Lopez-Amoros ML, Hernandez T, Estrella I (2006). Effect of germination on legume phenolic compounds and their antioxidant activity. J. Food Comp. Anal.

[5] Fernandez-Orozco R, Piskula MK, Zielinski H, Kozlowska H, Frias J, Vidal-Valverde C (2006). Germination as a process to improve the antioxidant capacity of Lupinus angustifolius L. var. zapaton. Eur.Food Res. Technol.

[6] Shams Ardekani MR, Hajimahmoodi M, Oveisi MR, Sadeghi N, Jannat B, Ranjbar AM, Gholam N, Moridi T (2011). Comparative antioxidant activity and total flavonoid content of Persian pomegranate (Punica granatum L.) cultivars. Iranian J. Pharm. Res.

[7] Cevallos-Casals BA, Cisneros-Zevallos L (2010). Impact of germination on phenolic content and antioxidant activity of 13 edible seed species. Food Chem.

[8] Chung SK, Osawa T, Kawakishi S (1997). Hydroxyl radical-scavenging effects of spices and scavengers from brown mustard (Brassica nigra). Biosci.Biotechnol. Biochem.

[9] Bahramiyan F, Ghiassi Tarzi B, Yaghmaei S, Bahonar A (2012). Extraction of plum phenolic compounds using different solvents - the antimicrobial effect of the extracts on the growth of E. coli , S. aureus. J. FoodTechnol. Nutr.

[10] Kamaliroosta L, Ghavami M, Gharachorloo M, Azizinezhad R (2011). Isolation of cinnamon extract and assessing its effect on the stability of sunflower oil. Iranian J. Nutr. Sci. Food Technol.

[11] Gharachorloo M, Ghavami M, Jamdar F Stabilising activities of acetone and ethanolic extracts of turmeric on canola and sunflower seed oils. J. Oxid. Commun.

[12] Firestone D (1990). Official methods of analysis of theAssociation of Official Analytical Chemists.

[13] Atienza J, Sanz M, Herguedas A, Alejos JA, Jimenez JJ (1998). Beta-carotene, alpha-tocopherol and gamma-tocopherol contents in dry legumes: influence of cooking. Food Sci. Technol. Int.

